# Loss of productivity among caregivers of dependent family members

**DOI:** 10.3934/publichealth.2025025

**Published:** 2025-04-11

**Authors:** Laura Gonzalo-Ciria, Marta Pérez De Heredia-Torres, María Isabel Vidal-Sánchez, María José López-de-la-Fuente, Elisa Bullón-Benito, Ana Poveda-García, Mariana Ortiz-Piña, María Cristina Ruiz-Garrós, Ana Gascón-Catalán

**Affiliations:** 1 Department of Physical Therapy, Occupational Therapy, Rehabilitation and Physical Medicine, Faculty of Health Sciences, Rey Juan Carlos University, Madrid, Spain; 2 Department of Physiatry and Nursing, Faculty of Health Sciences, University of Zaragoza, Zaragoza, Spain

**Keywords:** productivity, work performance, caregiving, informal caregivers, paid work, household, education, occupational satisfaction, quality of life, roles

## Abstract

**Background:**

Assuming the care of a dependent family member can constitute a health risk factor that significantly reduces the productivity of family caregivers, compromising their future and quality of life.

**Objective:**

Our purpose of this study was to investigate the productivity activities that are altered in the caregivers of a dependent family member and the impact this has on their satisfaction and quality of life.

**Methods:**

An analytical observational study was conducted for 500 caregivers of dependent family members. Sociodemographic data were collected for caregivers and their dependent family members. Carers' occupational performance and satisfaction were assessed using the Canadian Occupational Performance Measure (COPM), and quality of life was assessed using the World Health Organization - Quality of Life (WHOQOL-Bref). Comparison between groups was performed using the Chi-square test for qualitative variables. Pearson's correlation coefficient was used to assess the strength and direction of linear associations between numerical variables.

**Results:**

Most caregivers were women (72.3%, n = 364), with an average age of 60.3 ± 13.64 years. These individuals were providing care for a family member with severe dependency (69.7%, n = 348), classified as Grade III. Up to 38.2% (n = 191) of caregivers struggled with maintaining punctuality and consistency in their paid employment, and 25.6% (n = 128) of caregivers stopped or had difficulty working. In addition, 28.4% (n = 142) of caregivers had problems with household cleaning and tidying, 20.4% (n = 102) perceived that they neglected their other family members, and 18.6% (n = 93) of caregivers encountered problems attending courses and furthering their education. Women were more affected in terms of productivity. Moreover, performance and quality of life worsened as the number of productivity activities affected increased.

**Conclusions:**

Caring for a dependent family member has a considerable impact on the caregiver's productive activities, affecting their work performance, household management, and professional development, with a particularly marked impact on women. This caregiving role is also associated with a decrease in quality of life, which highlights the need for interventions to support caregivers in these areas.

## 1. Introduction

### 1.1. General considerations

Caring for chronically ill and/or dependent individuals at home is a demanding process that can place a great burden on the family caregiver [1],[2]. However, according to the Organization for Economic Cooperation and Development in its 2022 report [3], health services in Europe do not offer sufficient support to family caregivers, affecting even more countries such as Spain, where the family is the main source of assistance.

According to the Framework for Occupational Therapy Practice developed by the American Occupational Therapy Association, caregiving is considered an instrumental activity (activity to support daily life in the home and community) that involves providing care and supervisory activities [4]. It presents two categories of activities that are related to caring for a family member:

The act of caring for others (providing care to others, arranging or supervising formal care (by paid caregivers) or informal care (by family or friends) for others).
Parenting (providing care and supervision to support a child's developmental and physiological needs) [4].

However, there is a marked difference in caregiving when a family member has a disability and/or chronic illness because the caregiving role can arise suddenly without being chosen and, in most cases, without prior preparation. This situation, which often extends indefinitely, can have a significant impact on the quality of life of the caregiver [5].

In the context of parenting, caring for a child with a disability and/or chronic illness entails an additional burden that is continuously prolonged over time. Parenting, as well as most problems related to health and illness, are managed in the domestic space. Most parents are accustomed to taking on the care of their children single-handedly, based on the belief that raising their child with multiple disabilities could be a similar task to raising a child without a disability, later recognizing the hardship they faced in caring for their child alone [6].

Although several researchers have provided evidence of positive aspects of caregiving, including lower depression and higher life satisfaction rates, the role of caregiver of a dependent family member (CDFM) is highly stressful, impacting family organization [7],[8] and is associated with physical, psychological, and financial burdens that cause serious health and socio-family problems for the caregiver [9]–[11], affecting their quality of life [12]–[14].

This issue becomes even more relevant when we consider the increase in life expectancy. Although lifestyles can reduce the risk of fatal diseases, these changes do not alter the onset or progression of most chronic and/or degenerative diseases associated with dependency. These diseases are becoming more prevalent, last longer, require more care and are beyond the capacity of the current social care model, which is based on the role of the family and women in particular [15]. In parallel, social changes, such as the incorporation of women in the workplace, and changes in the traditional family model, among others, generate a series of social processes, where the number of dependent people increases, while the number of people available to care for them decreases, making it necessary to rethink care programs and policies [16]–[20].

The Canadian Occupational Performance Model distinguishes three domains of performance: Self-care, productivity, and leisure. Although caregiving for a dependent family member can affect all three domains of performance [21]–[23], in this study, we focused on productivity, understood as activities that typically occupy most of the day and contribute to economic maintenance, home and family maintenance, and personal or service development [24].

**Figure 1.**
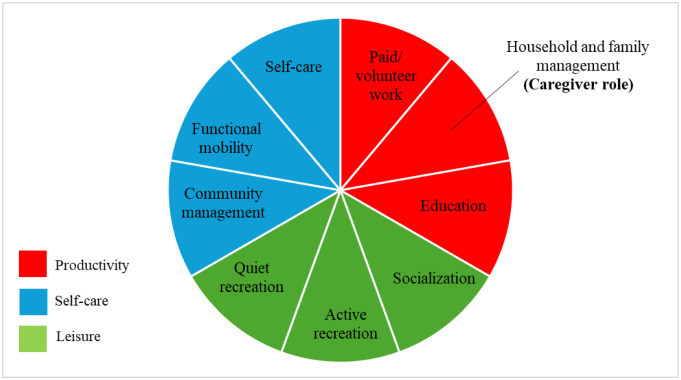
Performance areas according to the Canadian Occupational Performance Measure. Source: own elaboration based on the referenced literature.

In this model, we use the Canadian Occupational Performance Measure (COPM) as an instrument to analyze the affected performance activities of the individual, classifying productivity activities (PAs) as follows [25]:

Paid and/or volunteer work;
Household and family management (which includes care of a dependent family member);
Training activities (Figure 1).

Most researchers and programs consider caregiving as the main activity of CFDs, with their goals focused on improving the performance of their caregiving role [19] to ensure better care for family members. On the other hand, some researchers focus on the paid work of DFCs, highlighting how they experience substantial work interruptions and negative work performance outcomes. These researchers analyze the impact of caregiving in terms of economic losses or opportunity costs [26]–[28], which is essential for understanding the impact of caregiving in economic terms. However, both approaches fail to study productivity as a whole, as they do not consider household and family management and training activities, as suggested by the COPM (Figure 1), and therefore do not analyze all the productive activities affected in order to provide solutions for this population. Furthermore, they vaguely describe gender differences among caregivers [16],[18],[19], a necessary focus in caregiving-related studies.

### 1.2. Study purpose

Our aim of this study was to investigate the productive activities affected in CDFM, as a consequence of their caregiving role, and the relationship with the deterioration in their quality of life. Furthermore, we seek to emphasize the significance of CDFM productivity, offering insights that can inform the redesign of family care programs and the enhancement of social policies in this domain.

## 2. Methods

### 2.1. Materials

An observational study was conducted among caregivers and their dependent family members. Participants were recruited from 20 social-health institutions in the city of Zaragoza (Spain) that care for people with disabilities and/or chronic diseases and/or their caregivers, based on the following inclusion criteria:

Family caregiver aged 18 years or older and not receiving specific remuneration for the care provided.
The family member receiving care must reside in the city of Zaragoza (Spain), regardless of where the caregiver lives.
The caregiver must be the main caregiver, share the care with other caregivers, or collaborate occasionally in the care, for at least one year.
The family member being cared for suffers from a functional limitation of a physical, mental, intellectual, and/or sensory nature, derived from age and/or chronic illness, and may be dependent or only need supervision and/or support in activities of daily living on a long-lasting basis.

These criteria were proposed in the study based on the consulted literature and on the authors' professional experience in public and private administration in Spain.

### 2.2. Sample

A sample size of 377 persons was calculated with a maximum margin of error of 5% for a confidence level of 95%, and assuming maximum variance (p = q = 0.5). Finally, a sample of 500 people was obtained, with a margin of error of 4.3% at the 95% confidence level.

Given that access to caregivers was limited, convenience sampling was used. The sample size calculation enabled us to estimate the minimum number of participants to obtain statistically robust conclusions, optimizing the available resources and ensuring the internal validity of the study.

### 2.3. Instrumentation

To collect the data for this research, a questionnaire was created. It consisted of 95 questions divided into two sections:

Section for the family member receiving care: Demographic data and degree of dependency were collected according to the criteria of the Aragonese System of Care for Dependency of Aragon (Spain) [29].

Section for the CDFM: Caregiver demographics were collected, together with the two selected assessment tools:

*A) The Canadian Occupational Performance Measure (COPM) was used to assess the performance and occupational satisfaction of caregivers*.

The COPM is an instrument widely used by occupational therapists and multidisciplinary teams around the world. Its psychometric properties have been extensively studied with satisfactory results in different types of health care settings and among different types of users. A Spanish version is available from Curtin et al., 2016 [25], and the Spanish COPM provides satisfactory measurement properties as a client-centered instrument [30] and has demonstrated its flexibility and adaptability to different situations, clients, settings, and purposes related to family caregivers [30]–[33]. It is a client-centered outcome measure that facilitates the prioritization of individualized interventions. The patient lists a free-form list of performance problems that the patient is free to state qualitatively and the patient is then asked to quantify their performance, satisfaction, and importance in relation to the patients' responses.

Following the recommendations of the COPM, a checklist of potential difficulties that caregivers may experience was created to facilitate the administration of the COPM based on the literature [26],[34] and the professional experience of the researchers. The respondent identified the five most important activities affected by his or her role as a caregiver. In addition, each of the selected activities was rated on a scale of 1 to 10 to express their level of performance and satisfaction in the performance of these activities [25],[31]. These data were used to calculate the average performance and satisfaction of the population studied. The reliability analysis of the Canadian Occupational Performance Measure (COPM) in this study indicated good internal consistency, with a Cronbach's alpha of 0.849.

*B) WHOQOL-Bref (World Health Organization - Quality of life, 1998)*.

The WHOQOL-Bref, a quality-of-life assessment tool developed by the World Health Organization (WHO), has been translated into numerous languages for application in different cultural contexts and has been validated for the Spanish population [35],[36]. The WHOQOL-BREF is mainly used for epidemiological studies and clinical trials where quality of life is of interest, but not necessarily the main object of study. It focuses on the subject's “perceived” quality of life. Therefore, it is not expected to measure the symptoms, the disease or the condition itself, but the effects of the disease and health interventions on quality of life. In this way, it aims to complete the triad “person-environment-occupation” in order to analyze the occupational performance of caregivers. It is currently one of the most widely used in research and has been used in different investigations of caregivers [37].

It consists of 26 questions that are self-rated on a scale from 1 to 5, where 1 is the worst condition and 5 is the highest rating. It provides a profile of QoL as perceived by the person across four domains: Physical health, psychological health, social relationships, and environment, that are not always included in other questionnaires [38],[39].

It was chosen for this research because it is a clear, simple, and easy to complete tool. The WHOQOL-Bref questionnaire demonstrated good internal consistency in this study, with a Cronbach's alpha of 0.937.

The questionnaire was administered in two formats: On paper or via an online form. The questionnaires were distributed and collected in person at the collaborating centers and were administered in person in cases where assistance was needed to complete the questionnaire.

The study lasted 14 months, from the time the managers of the centers that care for dependent persons or family members were contacted until all interviews were conducted and all questionnaires were collected. The time required to complete the questionnaire was approximately 30 minutes.

### 2.4. Data analyses

Qualitative variables were described by absolute (n) and relative (%) frequencies and mean and standard deviation (SD) for quantitative variables. Comparison between groups was performed using the Chi-square test (qualitative variables). Specifically, it was used to determine if there were significant differences in the affected productivity activities between male and female family caregivers. To assess the relationship between variables, Spearman's correlation coefficient was calculated for ordinal variables. In the case of quantitative variables, Pearson's correlation coefficient was used after verification of compliance with the assumptions of normality using the Kolmogorov-Smirnov test. The Pearson's correlation coefficient was used to analyze whether there was a linear relationship between the quantitative variables: number of activities affected at work, household management, and leisure with occupational performance and satisfaction and QoL. Statistical analysis was performed with SPSS 23.0 for Windows. The differences considered statistically significant were those with a p < 0.05.

### 2.5. Ethical data

The study was approved by the Research Ethics Committee of the Community of Aragón (PI17/0039) and was conducted in accordance with the ethical principles of the World Medical Association's Declaration of Helsinki. All participants signed an informed consent form, and the necessary measures were taken to guarantee the privacy and confidentiality of their personal data. In addition, all organizations involved in the study signed an agreement and gave their permission to conduct the research.

## 3. Results

The sample included 500, mostly female, CDFMs (72.8%; n = 364), with a mean age of 60.3 ± 13.64 years. Three quarters lived with the family member for whom they provided care (n = 377). Only 38.4% (n = 192) were working and most had university or higher education (63.4%; n = 311). A total of 28.4% (n = 142) were retired and more than one third of the caregivers had illnesses that made it difficult for them to care for their relative (38.8%; n = 108). They had provided care for a mean of 13.2 ± 12.05 years, spent almost 15 hours per day on caregiving, and had 4 ± 4.65 hours per day free from caregiving responsibilities (Table 1).

**Table 1.** Sociodemographic and caregiving characteristics of the family caregiver.

Characteristic	Mean (SD); range	n (%)
Participants		
Age, years	60.3 (±13.64); 18–96	
Gender		
Female		364 (72.8)
Male		136 (27.2)
Marital status		
Married/in a relationship		380 (76)
Single/Widowed/Separated		120 (24)
Relationship		
Son/daughter		168 (33.6)
Spouse, partner		144 (28.8)
Parent		153 (30.6)
Others		35 (7)
Educational level		
University Studies		158 (32.2)
Secondary Education		153 (31.2)
Primary Education		146 (29.8)
Without Compulsory Education		33 (6.7)
Employment status		
Actively working		192 (38.4)
Inactive		166 (33.2)
Retired		142 (28.4)
Full time job		136 (27.2)
Household chores		96 (19.2)
Unemployed		46 (9.2)
Reduced workday (less than 4h/day)		41 (8.2)
Disability/pensioner		24 (4.8)
Temporary work		8 (1.6)
Flexible work hours		7 (1.4)
Years of care/supervision/support	13.22 (12.05)	
Type of caregiver		
Primary Caregiver		365 (73)
Co-responsible caregiver (equal care sharing with another family member)		112 (22.4)
Collaborative caregiver (helps provide occasional care)		23 (4.6)
Number of family members cared for		
Sole caregiver for a dependent family member		357 (71.4)
Cares for more family members		143 (28.6)
Care provided		
Monitoring, control		443 (88.6)
Emotional support		398 (79.6)
Instrumental activities of daily living		361 (72.2)
Basic activities of daily living		280 (56)
Frequency of care		
Every day of the week, 24 hours a day		99 (19.8)
Every day, except for the hours when the family member is at the center where he or she usually goes and/or after the caregiver's workday		259 (51.8)
Occasional supervision		144 (28.8)
Nº of hours of care on a normal day	14.5 hours (8.54)	
Nº of hours off without obligations on a normal day	4 hours (4.65)	
Presence of diseases that hinder care		108 (38.6)
Participation in self-help groups		137 (27.4)
Lives with the family member that is being cared for		377 (75.4)

Of the 500 family members cared for, most were women (55.8%; n = 279), had mixed disabilities (69.8%; n = 349), and had high dependency (69.7%; n = 348) (Table 2).

**Table 2.** Characteristics of the family members receiving care.

Characteristic	Mean (SD); range	n (%)
Participants		
Age, years	61 (28.2); 2–99	
Disability		
Intellectual and developmental disability		335 (67)
Mental/cognitive disability		279 (55.8)
Sensory disability		256 (51.2)
Physical disability		253 (50.6)
Frequency of assistance		
Once a day		27 (5.5)
2–3 times a day		119 (23.7)
On demand		18 (3.6)
Continuous		336 (67.2)
Level of dependency		
I (moderate)*		29 (5.7)
II (severe)**		123 (24.6)
III (high dependency)***		348 (69.7)

Note: *The family member needs help with basic activities of daily living (BADL) once a day; **The family member needs help to perform several ADLs, two or three times a day, without permanent support from the caregiver; ***The family member needs help to perform several ADLs, several times a day or has total loss of physical, mental, intellectual, or sensory autonomy; and he/she needs indispensable and continuous support from another person.

Table 3 details the PAs affected, divided into three sub-areas: Paid/voluntary work, household and family management, and education. Regarding paid/voluntary work, 38.2% (n = 191) of caregivers had difficulty maintaining punctuality and consistency in paid work, and 25.6% (n = 128) of caregivers have left work or have difficulty working. Regarding household management, 31.7% (n = 156) had to move or adapt their home and 28.4% (n = 142) of caregivers had problems with cleaning and tidying the home. In addition, 20.4% (n = 102) perceived that they were neglecting the rest of their family. Regarding educational activities, 18.6% (n = 93) of caregivers had problems attending courses and expanding their education. It should be noted that only 8% (n = 44) of the sample indicated that they had problems attending mutual support groups for family members.

Significant differences were found between the PAs and the sex of the caregivers: Women had more difficulty working and/or had to stop working at a higher rate (30.9%; n = 109) compared to men (11.4%; n = 15). They also had twice as much difficulty finding or changing jobs as men (14.7%; n = 52 vs. 7.6%; n = 10). Similarly, women had more difficulty volunteering (9.1%; n = 32 vs. 3.8%; n = 5) and felt more unable to take care of their families than men (22.7%; n = 80 vs. 14.4%; n = 19). No significant differences were found between men and women regarding the educational or training activities affected (Table 3).

**Table 3.** Productivity activities in which the family caregiver has difficulty and their relation to gender.

Variables	Caregivers N (%)	Sex		χ^2^
		Men	Women	p
Paid/volunteer work				
Difficulty working/ has stopped working	124 (25.6)	15 (11.4)	109 (30.9)	<0.001***
Work-related problems and conflicts	15 (3.2)	1 (0.8)	14 (4)	0.069
Holding positions of higher qualification	32 (6.8)	6 (4.5)	26 (7.4)	0.266
Volunteering at least once a week	37 (7.6)	5 (3.8)	32 (9.1)	0.051
Participating and getting involved in associations, groups	66 (13.2)	15 (11.4)	51 (14.4)	0.378
Seeking or changing employment	62 (12.8)	10 (7.6)	52 (14.7)	0.036*
Reduction of working hours	68(13.8)	15 (11.4)	53 (15)	0.303
Has changed jobs	19 (3.8)	2 (1.5)	17 (4.8)	0.095
I have never worked due to the caregiving	14 (2.8)	1 (0.8)	13 (3.7)	0.087
Attending interviews, meetings ...	55 (11.4)	15 (11.4)	40 (11.3)	0.992
Learning new tasks	44 (9.2)	7 (5.3)	37 (10.5)	0.077
Relating with my coworkers	40 (8.8)	11 (8.3)	29 (8.2)	0.966
Being punctual and consistent at work	183 (38.2)	48 (36.4)	135 (38.2)	0.704
Difficulty in all the activities I would like to perform	79 (16.8)	17 (12.9)	62 (17.6)	0.214
Household and family management				
Changing homes because of the caregiving	38 (7.6)	9 (6.8)	29 (8.2)	0.61
Adapting my home	117 (23.6)	29 (22)	88 (24.9)	0.498
Meal planning, food shopping, food preparation...	76 (15.4)	27 (20.5)	49 (13.9)	0.076
Cleaning and keeping my home tidy	142 (28.4)	40 (30.3)	102 (28.9)	0.762
Washing and ironing clothes	119 (24)	30 (22.7)	89 (25.2)	0.571
Replacing or repairing small things	67 (13.4)	15 (11.4)	52 (14.7)	0.339
Taking care of my relationship with my partner	24 (4.8)	7 (5.3)	17 (4.8)	0.826
Being able and/or knowing how to care for my family member	69 (14.2)	22 (16.7)	47 (13.3)	0.347
Neglecting the rest of my family	99 (20.4)	19 (14.4)	80 (22.7)	0.044*
Difficulty in all the activities I would like to perform	25 (5)	7 (5.3)	18 (5.1)	0.928
Education/training				
Studying for a degree	37 (7.4)	13 (9.8)	24 (6.8)	0.26
Take courses, improving my training, going to an academy, etc.	92 (18.6)	25 (18.9)	67 (19)	0.992
Attending classes full or part time	32 (6.6)	9 (6.8)	23 (6.5)	0.905
Taking exams, competitive examinations	22 (4.6)	6 (4.5)	16 (4.5)	0.995
Relating with my classmates	14 (2.8)	5 (3.8)	9 (2.5)	0.468
Mutual support groups	44 (8)	14 (10.6)	30 (8.5)	0.472
Difficulty in all the activities I would like to perform	40 (8)	12 (9.1)	28 (7.9)	0.680

Note: *p < 0.05; **p < 0.01; ***p < 0.001.

Caregivers revealed a performance of 3.77 (2.18) and satisfaction of 5.02 (2.69) on a scale of 0–10. The QoL scores for physical health were 56.39 (19.49), psychological health was 54.75 (18.52), social relationships was 50.1 (20.84), and environment was 54.31 (15.51) (on a scale of 0–100) (Table 4).

**Table 4.** Evaluation of performance, satisfaction, and quality of life of caregivers.

Variables	Mean	SD
Performance	3.77	2.18
Satisfaction	5.02	2.69
Health Physical	56.39	19.49
Health Psychological	54.75	18.52
Social Relationships	50.1	20.84
Environment	54.31	15.51

After analyzing productivity and its relationship with performance, satisfaction, and QoL, it was observed that as the number of productivity activities affected increased, performance and QoL decreased, with the exception of educational activities. No correlation was found with the level of satisfaction (Table 5).

The number of affected activities in patients related to paid work ranged from 0 to 10 (mean = 1.57, SD = 1.92); from 0 to 8 in household management (mean = 1.52, SD = 1.77); and from 0 to 5 in education/training (mean = 0.40, SD = 0.87).

**Table 5.** Correlation between the number of productivity activities affected and occupational performance, together with satisfaction scores and quality of life (physical, psychological health, social relationships, and environment).

Variables	Performance	Satisfaction	Quality of life			
			Physical Health	Psychological Health	Social Relationships	Environment
Paid work	−0.112*	−0.086	−0.171**	−0.124**	−0.199**	−0.217**
Household management	−0.178**	−0.053	−0.307**	−0.247**	−0.268**	−0.292**
Education	−0.106	−0.056	−0.067	0.029	−0.017	−0.037

Note: r = Pearson's correlation coefficient, *p < 0.05; **p < 0.01.

## 4. Discussion

We take a comprehensive approach to a public health problem by focusing not only on the growing number of people with dependency, but also on their family caregivers [23].

For the first time, PAs and their relationship with the performance, occupational satisfaction, and QoL of a large and diverse population of CDFM are analyzed. In addition, this study offers a perspective that incorporates both gender and occupational approaches, enabling a deeper understanding of the dynamics faced by caregivers in their daily work.

Most published studies encompass solely the role of the caregiver for the improvement of the quality of life of CDFM [39]. This research contributes to the analysis of productivity as a whole, giving value to the rest of the productive activities affected as a consequence of caregiving. Thus, following the COPM guidelines, productivity is divided into three subareas: Paid and/or voluntary work, household and family management, and education.

### 4.1. Paid and/or volunteer work

Caregiving has a negative impact on the work performance of CDFM, as they miss a significant amount of work and experience a reduction in productivity due to their caregiving responsibilities [26],[41].

Our research shows similar results. The novelty of this study is its demonstration of which activities are affected in a heterogeneous population of family members. Our findings indicate that approximately 25% of our CDFM experience challenges in maintaining their professional roles or have even ceased working to prioritize their caregiving responsibilities. Consistency at work and punctuality stand out as the activities most affected among caregivers (one in three caregivers); one in four caregivers have difficulty going to work and/or give up their professional activity. In addition, other productivity activities are limited, such as advancing in the world of work, obtaining more qualified positions, maintaining work-related social relationships, attending interviews, meetings, learning new tasks, and looking for or changing jobs. They also perceive problems and conflicts at work, which may lead to reducing or abandoning work. In addition, caregivers have difficulty volunteering, which is understandable given their limited free time and perceived unimportance of their role [7].

The challenge of reconciling work and family responsibilities is further compounded when one assumes the role of a caregiver for a dependent individual. The time invested in medical visits, diagnostic tests, and treatments for the care recipient inevitably results in a compromise of work schedule fulfillment and job stability [35], and therefore affecting performance in this area.

A comparison of our results with those of other researchers is challenging due to the lack of studies that quantify and analyze the PAs of CDFM. Nevertheless, further research on this subject is required in order to gain insight into the impact of caregiving on the productivity of CDFM. This could inform the reorientation of social programs and policies towards a model of co-responsibility in caregiving. The act of caring for a family member should be a voluntary undertaking and should not be subject to pressure from a social and healthcare system that fails to meet the needs of individuals with dependency [40].

### 4.2. Household and family management

Unlike most researchers who focus on caregivers, and in line with Agulló Cantos et al. [7], we present the caregiver role as just one more facet, within a diversity of roles that anyone can play, such as being a student, worker, volunteer, family member, homemaker, among others [42], and not as the only productive role. According to the COPM, caregiving is within the productivity domain, specifically within the subdomain of household and family management [25]. Caregiving is a task that depends on the needs of the dependent person and may require a small amount of work, or it may be a large task that lasts a long time and takes up a large part of the caregiver's routine. Thus, caregivers tend to organize their lives according to the person they are caring for [34]. They often have difficulties such as keeping their home tidy and clean; they need help with laundry and sewing; and they have difficulty preparing meals and replacing or repairing technical problems in their home. Some CDFM have even moved or adapted their home to meet the needs of their family member to improve the quality of care. Regarding the role of caregiver, they have difficulties in caring for their family member and in reconciling caregiving with their family and couple life. These results are in line with those obtained by other authors who confirm the existence of a higher frequency of family dysfunction and a feeling of inadequate social support among the group of caregivers [8],[13].

Even though one in four caregivers in our study participated in family peer support groups, a considerable proportion of them admitted that they lack the knowledge or resources to care for their family member effectively. Most caregiver programs focus primarily on the caregiver's role as a caregiver, neglecting all other roles. An example of this are the programs aimed at caregivers of patients with progressive disease focusing on topics such as information, dealing with emotions, coping skills, and communication [34]. Although some studies [43] show the effectiveness of these programs, our findings support authors such as Ortiz-Mallasén et al. [14] who question its effectiveness with informal caregivers of older adults with dementia. According to our findings, it is possible that the solution lies not only in strengthening the caregiver's competencies as a caregiver, but also in understanding the caregiver's other roles, providing resources for household management, and caring for the rest of the family. More research is certainly needed in this regard.

### 4.3. Education

According to our results, caregivers' personal and professional training can also be affected by caregiving. Our caregivers presented difficulties in taking courses/extending their education and studying languages, experiencing difficulties in attending face-to-face classes, taking exams and competitive examinations, and studying for an academic degree. These findings coincide with those obtained by García et al. [44], who affirm that the major PAs affected in caregivers are the ability to obtain paid work and to continue studying. Although the role of student is rated as important for men and very important for women caregivers [7], almost one fifth of the caregivers in our study have difficulty furthering their education. This suggests that training may be one of the first activities they abandon or do not even consider because of their situation. However, there is a clear lack of studies analyzing how caregiving affects caregiver training.

### 4.4. Gender differences in productivity

Our results reveal that women face significantly greater work-related and emotional challenges than men. Women are three times more likely than men to quit their jobs and twice as likely to have difficulty finding or changing jobs. In addition, women feel more neglected by their families than men and have more difficulty volunteering. This is in line with other research [27] revealing that women are more likely to request reduced working hours and leave of absence to care for their families and give up more of their jobs, since they have lower salaries than men, as a result of the wage gap [46]. The major reasons cited in the literature correspond to the caregivers' constant attention to their family members, especially mothers to their children, and continual accompaniment to medical appointments, diagnostic tests and treatments; the caregivers' perception and/or conviction that they are indispensable in the care of their children and that it is very compromising to delegate complex care to another person [6],[34],[41]. These causes reflect the traditional family model that persists in some countries, such as Spain, which reveals marked gender differences further jeopardizing women's futures [46] and QoL through its direct link to health [8]. These differences become even more important when compared to general population studies, where men show an increasingly stronger relationship between health problems and presenteeism than women [40],[47].

### 4.5. Performance, satisfaction and QoL

Our data showed that productivity was affected in all its subdomains, which was related to lower performance and quality of life (QoL). According to our results, as the number of affected productivity activities increases, the performance and QoL of CDFMs decreases. These data would agree with WHO philosophies, such as that of the Ottawa Charter for Health Promotion [48] and Wilcock AA theory [49], where performance and participation in individually and socially valued meaningful occupations are highlighted as factors that benefit people's health and well-being. Considering the results of this research coupled with the results of other research quantifying the high economic and opportunity costs suffered by caregivers [27],[28], it is easy to understand how the future and quality of life of CFMs are compromised.

There are many studies that associate caregiving with a greater impact on QoL [9],[21],[50],[51]; however, to the best of our knowledge, we are the only ones to analyze PAs in depth, distinguishing the role of caregiver from other roles that affect caregivers' productivity and, therefore, their quality of life. Our results are difficult to compare with other research, as there are few studies that use the COPM with family caregivers. Studies such as those by Mulcahey et al. [32], Gatta et al. [31] use the COPM in a homogeneous sample of caregivers but do not describe the affected performance areas (PAs) in family caregivers, instead grouping them into a single area of occupation. However, all studies agree with our research in obtaining low levels of performance and occupational satisfaction among their caregivers, despite studying different populations of family caregivers. Our research adds to the literature by providing a comprehensive approach to caregiver performance and satisfaction, presenting a heterogeneous sample of caregivers composed of all types of family caregivers regardless of the disability and/or chronic illness of the family member they care for. Additionally, it describes the affected PAs that give rise to performance and satisfaction problems, to be addressed in future interventions. We believe further research is needed to better understand how caregiving impacts family caregivers' productivity. Such insights could help reshape social programs and policies to promote shared caregiving responsibilities.

Considering the improvement of the caregiver role as the main goal of programs targeting CDFM could be a big mistake. Most programs prioritize efforts to strengthen the caregiver role while ignoring other roles such as worker, student, homemaker, to improve the quality of care and ensure the quality of life of family members. Some researchers have focused on how improving the quality of life of the caregiver can improve the quality of life of the family member receiving care [52]. However, these approaches once again relegate the family caregiver to the background in the face of a health situation that deserves equal priority for both dependent persons and their relatives. Based on our findings, we believe that it is necessary to work on all sub-areas of productivity, including paid/voluntary work and training activities, as well as home and family management. This could improve the quality of life of CDFM and the effectiveness of programs for this population, the effectiveness of which has been debated in the literature [14]. In addition, it is essential to develop new lines of research that work along these lines.

Regarding occupational satisfaction, we have not found any correlation with the number of PAs affected, nor have we found any literature addressing this issue. The concepts of the theory developed by Wilcock [49] suggest that depriving people of the opportunity to participate in meaningful activities can affect their well-being and QoL. This could provide an explanation for justify caregivers' satisfaction in terms of quality rather than the quantity of affected productive activities experienced by those with CDFM who are compelled to leave their employment or other significant productive roles in their personal histories in order to care for a family member. This theory posits that health is a state of equilibrium between physical, mental, and social well-being, achieved through the pursuit of personally and socially valued meaningful occupations. In contrast, it suggests that the lack of opportunity for these activities and the lack of social recognition experienced by caregivers in the arduous task of caring for their family member contribute to a state of imbalance [51]. In this manner, we would be discussing occupational injustice as an additional factor to be considered alongside other studies that examine care in terms of opportunity costs [53],[54].

Therefore, the family caregiver can be described as a citizen deprived of opportunities with a vulnerable QoL, especially women, which is why the incorporation of the occupational approach together with the gender approach in research is key because of its direct relationship with health. Studies such as this one could be key to reorienting social programs and policies that favor the opportunity to perform their productive roles, and not only the role of caregiver, prioritizing co-responsibility in caregiving.

### 4.6. Methodological considerations/limitations

Our results should be treated with caution because, although the sample size was adequate, the population was limited to a single region in Spain, which limits the generalizability of the findings to other geographical or sociocultural contexts. In addition, it should be noted that nearly one-third of the participants in this study were retired, which may have influenced the results related to paid work activities, suggesting that these percentages may increase in the working population.

We focused on the productivity of CFDs. However, we should not forget the other occupations in this population.

Variables such as caregiver age, cohabitation with the family member, community resources, and social support that were not analyzed in this study may have influenced the results. However, we chose to focus on caregivers to address their needs from an occupational and gender perspective.

## 5. Conclusions

Caregivers of family members with disabilities and/or chronic illnesses, especially women, experience a decline in their productivity, especially in terms of consistency and punctuality in paid work and, in some cases, even leave their jobs. This situation has a negative impact on their work performance and quality of life. Recognizing the role of caring for a dependent family member as the only productive role of the caregiver is a conceptual error that should be reconsidered. It is therefore essential to review social programs and policies related to caregiving in order to address the multiple roles of caregivers in a comprehensive manner.

## Use of AI tools declaration

The authors declare they have not used Artificial Intelligence (AI) tools in the creation of this article.

## References

[1] Tejero-Aranguren J, García Del Moral R, Poyatos-Aguilera ME (2024). Family burden after critical illness: the forgotten caregivers. Med Intensiva (Engl Ed).

[2] Sánchez Bárcenas RA, López Hernández D, Brito-Aranda L (2024). Factors associated with caregiver burden in primary caregivers of older adults with type2 diabetes. Aten Primaria.

[3] OECD (2022). Evolving Family Models in Spain: A New National Framework for Improved Support and Protection for Families.

[4] Boop C, Cahill SM, Davis C (2020). Occupational therapy practice framework: Domain and process fourth edition. Am J Occup Ther.

[5] Saletti-Cuesta L, Tutton E, Langstaff D (2018). Understanding informal carers' experiences of caring for older people with a hip fracture: a systematic review of qualitative studies. Disabil Rehabil.

[6] Roca Roger M, Úbeda Bonet I, García Viñets L (2012). Padres que cuidan a sus hijos con plurisdiscapacidad: estudio cualitativo sobre el cuidado y sus consecuencias. Siglo Cero: Revista Española sobre Discapacidad Intelectual.

[7] Agulló Cantos JM, Paredes-Carbonell JJ, García-Alandete J (2019). Roles e intereses en familiares cuidadores de personas diagnosticadas con enfermedad de Alzheimer. Revista TOG.

[8] Castellanos L (2022). La carga del cuidado: repercusiones en la salud de las cuidadoras de personas con discapacidad. MUSAS. Revista de Investigación en Mujer, Salud y Sociedad.

[9] Fernández-Ávalos MI, Pérez-Marfil MN, Ferrer-Cascales R (2020). Quality of Life and Concerns in Parent Caregivers of Adult Children Diagnosed with Intellectual Disability: A Qualitative Study. Int J Environ Res Public Health.

[10] Swartz K, Collins LG (2019). Caregiver Care. Am Fam Physician.

[11] Hazzan AA, Follansbee P, Dauenhauer J (2020). Relationship between family caregiver quality of life and the care provided to people living with dementia: protocol for a mixed methods study. AIMS Public Health.

[12] Stragapede E, Petricone-Westwood D, Hales S (2023). Patient quality of life and caregiver experiences in ovarian cancer: How are they related?. Qual Life Res.

[13] Young K, Cashion C, Ekberg S (2023). Quality of life and family functioning soon after paediatric brain tumour diagnosis: A cross-sectional observational study. Eur J Oncol Nurs.

[14] Ortiz-Mallasén V, Claramonte-Gual E, Cervera-Gasch Á (2021). Evaluation of the effectiveness of an intervention program in family caregivers of dependent persons, in the primary health care system. Aten Primaria.

[15] Stathopoulou A, Fragkiadakis GF (2023). Assessment of psychological distress and quality of life of family caregivers caring for patients with chronic diseases at home. AIMS Public Health.

[16] Bagatell N, Lamarche E, Klinger L (2023). Roles of Caregivers of Autistic Adults: A Qualitative Study. Am J Occup Ther.

[17] Castilblanco AC (2023). Las políticas de cuidado en algunos países de América Latina. Una mirada feminista. Ánfora.

[18] Letrondo PA, Ashley SA, Flinn A (2023). Systematic review of arts and culture-based interventions for people living with dementia and their caregivers. Ageing Res Rev.

[19] Santana E, Mendes F, Bernardo J (2023). Difficulties in Caring for the Older Adults: Perspective of Brazilian and Portuguese Caregivers. Nurs Rep.

[20] Gómez ÁM, Navarro JR (2019). Tendencias demográficas a escala mundial y sus repercusiones en la provisión de cuidado a las personas mayores dependientes. Actas de coordinación sociosanitaria.

[21] Davy G, Barbaro J, Unwin K (2024). Leisure, community, workforce participation and quality of life in primary and secondary caregivers of autistic children. Autism Res.

[22] Huang Y, Hu J, Xie T (2023). Effects of home-based chronic wound care training for patients and caregivers: A systematic review. Int Wound J.

[23] Guato-Torres P, Mendoza-Parra S (2022). Autocuidado del cuidador informal de personas mayores en algunos países de Latinoamérica: Revisión descriptiva. Enfermería: Cuidados Humanizados.

[24] McColl MA, Law MC, Debra S (2015). Theoretical Basis of Occupational Therapy (3rd ed.).

[25] Curtin M, Egan M, Adams J (2016). Occupational Therapy for People Experiencing Illness, Injury or Impairment E-Book (previously entitled Occupational Therapy and Physical Dysfunction): Occupational Therapy for People Experiencing Illness, Injury or Impairment E-Book (previously entitled Occupational Therapy and Physical Dysfunction).

[26] Keita Fakeye MB, Samuel LJ, Drabo EF (2023). Caregiving-Related Work Productivity Loss Among Employed Family and Other Unpaid Caregivers of Older Adults. Value Health.

[27] Ganapathy V, Graham GD, DiBonaventura MD (2015). Caregiver burden, productivity loss, and indirect costs associated with caring for patients with poststroke spasticity. Clin Interv Aging.

[28] Sruamsiri R, Mori Y, Mahlich J (2018). Productivity loss of caregivers of schizophrenia patients: a cross-sectional survey in Japan. J Ment Health.

[29] Aragon Government (2008). Sistema Aragonés de Atención a la Dependencia: guía informativa [Internet].

[30] Capdevila E, Portell M, Penelo E (2024). Propiedades de medición del COPM español en pacientes hospitalizados en rehabilitación de adultos mayores. Revista escandinava de terapia ocupacional.

[31] Gatta FD, Fabrizi E, Giubilei F (2022). Caregivers' Profiles Based on the Canadian Occupational Performance Measure for the Adoption of Assistive Technologies. Sensors (Basel).

[32] Mulcahey MJ, Gerhardt N, Alpajora B (2022). Coaching-in-Context With Informal Maternal Care Partners of Children With Spinal Cord Injury. Top Spinal Cord Inj Rehabil.

[33] Verkerk GJ, Wolf MJ, Louwers AM (2006). The reproducibility and validity of the Canadian Occupational Performance Measure in parents of children with disabilities. Clin Rehabil.

[34] de Wit J, Schröder CD, El Mecky J (2019). Support needs of caregivers of patients with amyotrophic lateral sclerosis: A qualitative study. Palliat Support Care.

[35] Lucas-Carrasco R (2012). The WHO quality of life (WHOQOL) questionnaire: Spanish development and validation studies. Qual Life Res.

[36] Benitez-Borrego S, Guàrdia-Olmos J, Urzúa-Morales A (2014). Factorial structural analysis of the Spanish version of WHOQOL-BREF: an exploratory structural equation model study. Qual Life Res.

[37] Shefeek SK, Joy TM, Olickal JJ (2024). Caregiver burden and quality of life in palliative care: cross-sectional study. BMJ Support Palliat Care.

[38] Salinas-Rodríguez A, Manrique-Espinoza BS, Montañez-Hernández JC (2022). Mediator effect of caregiver burden on the association between disability and quality of life among older adults. Salud Publica Mex.

[39] World Health Organization (2012). WHOQOL: Measuring quality of life.

[40] Zarzycki M, Vilchinsky N, Bei E (2024). Cross-country variations in the caregiver role: evidence from the ENTWINE-iCohort study. BMC Public Health.

[41] Martsolf GR, Kandrack R, Rodakowski J (2020). Work Performance Among Informal Caregivers: A Review of the Literature. J Aging Health.

[42] Kielhofner G (2002). A model of human occupation: Theory and application.

[43] Bernabéu-Álvarez C, Lima-Rodríguez JS, Lima-Serrano M (2022). Effect of support groups on caregiver's quality of life. Fam Process.

[44] García NAH, Campos AO, Bolaņos C (2018). Occupational Performance and Satisfaction of the Informal Primary Caregivers of Patients with Activity Limitations. RICS Revista Iberoamericana de las Ciencias de la Salud.

[45] Picatoste X, Mesquita A, González-Laxe F (2023). Gender wage gap, quality of earnings and gender digital divide in the European context. Empirica (Dordr).

[46] Rubinich G, Lopes Campos B, Souza Ramos LC (2021). ¿Promoviendo la igualdad de género? Los avances del G20 y el Women20 para erradicar la brecha economica. Revista SAAP.

[47] Ozawa S, Monma T, Tsuchida M (2024). Health Problems Related to Presenteeism Among Japanese Employees. J Occup Environ Med.

[48] World Health Organization Regional Office for Europe (‎1986)‎ Ottawa Charter for Health Promotion, 1986.

[49] Wilcock A, Hocking C (2024). An occupational perspective of health, Routledge.

[50] Lee K, Yefimova M, Puga F (2021). Gender Differences in Caregiver Burden Among Family Caregivers of Persons With Dementia. J Gerontol Nurs.

[51] Leyva-López A, Rivera-Rivera L, Márquez-Caraveo ME (2022). Estudio de la calidad de vida en cuidadores familiares de personas con discapacidad intelectual. Salud Publica Mex.

[52] Hazzan AA, Dauenhauer J, Follansbee P (2022). Family caregiver quality of life and the care provided to older people living with dementia: qualitative analyses of caregiver interviews. BMC Geriatr.

[53] Farré M, Kostov B, Haro JM (2018). Costs and Burden Associated With Loss of Labor Productivity in Informal Caregivers of People With Dementia: Results From Spain. J Occup Environ Med.

[54] Jacobs JC, Van Houtven CH, Tanielian T (2019). Economic Spillover Effects of Intensive Unpaid Caregiving. Pharmacoeconomics.

